# Impact of Glycemic Control After Reperfusion on the Incidence of Acute Kidney Injury Following Living Donor Liver Transplantation: A Propensity Score-Matched Analysis

**DOI:** 10.3390/medicina61081325

**Published:** 2025-07-23

**Authors:** Yeon Ju Kim, Hye-Mee Kwon, Yan Zhen Jin, Sung-Hoon Kim, In-Gu Jun, Jun-Gol Song, Gyu-Sam Hwang

**Affiliations:** Department of Anesthesiology and Pain Medicine, Asan Medical Center, University of Ulsan College of Medicine, 88 Olympic-ro 43-gil, Songpa-gu, Seoul 05505, Republic of Korea; yjans@amc.seoul.kr (Y.J.K.);

**Keywords:** acute kidney injury, hyperglycemia, hypoglycemia, liver transplantation, renal insufficiency, chronic, propensity score

## Abstract

*Background and Objectives:* Glucose instability has been established to be related to postoperative morbidity and mortality in liver transplantation. To date, the impact of maintaining optimal blood glucose (BG) levels on the incidence of acute kidney injury (AKI) following liver transplantation (LT) remains unclear. This study aimed to determine the impact of optimal BG level after reperfusion (REP BG) on the incidence of AKI after living donor LT (LDLT). *Materials and Methods:* This study retrospectively reviewed 3331 patients who underwent LDLT between January 2008 and December 2019. Patients were divided into optimal (110 mg/dL < BG < 180 mg/dL) and non-optimal (BG < 110 mg/dL or >180 mg/dL) REP BG groups. Multivariable logistic regression analysis was performed to assess factors associated with AKI. Propensity score matching (PSM) was used to compare the incidence of AKI, AKI severity, and progression to chronic kidney disease (CKD) between the groups. *Results:* The incidence of AKI was 66.7%. After PSM, patients in the optimal REP BG group showed a lower incidence of AKI (66.5% vs. 70.6%, *p* = 0.032). Multivariable logistic regression analysis showed that the non-optimal REP BG group was independently associated with a higher risk of AKI (odds ratio [OR], 1.21; 95% confidence interval [CI], 1.02–1.45; *p* = 0.037) compared to the optimal group. Similarly, the risks of severe AKI (OR, 1.32; 95% CI, 1.11–1.58; *p* = 0.002) and progression to CKD (OR, 1.19; 95% CI, 1.01–1.41; *p* = 0.039) were significantly higher in the non-optimal group after PSM. *Conclusions*: Maintenance of an optimal REP BG was associated with a significantly lower incidence of AKI and a reduced risk of progression to CKD within 1 year after LDLT.

## 1. Introduction

Previous studies have established the detrimental effect of glucose instability, including hyper- and hypoglycemia, on postoperative outcomes in various fields, particularly in cardiac surgery and intensive care unit settings [1,2,3]. Glucose instability has been frequently observed in patients undergoing liver transplantation (LT) [4]. This can be attributed to factors such as insulin resistance, surgical stress, and the onset of gluconeogenesis after reperfusion of the newly transplanted graft [4,5,6]. Hyperglycemia is associated with increased mortality and an increased incidence of graft rejection and surgical site infection following LT [7,8]. Thus, in addition to the effects of hyperglycemia, the effect of hypoglycemia must also be considered, given its association with adverse outcomes [9].

Acute kidney injury (AKI) is a common and critical complication of LT [10] that results in prolonged duration of hospital stay, increased morbidity, and mortality [11,12]. The etiology of AKI following LT is multifactorial [13,14]; however, perioperative hyper- and hypoglycemia have been identified as potential risk factors for the incidence of AKI postoperatively [15,16]. A recent study revealed that increased glucose variability, rather than hyper- and hypoglycemia alone, is associated with the incidence of AKI following LT [17]. The discrepancies between the findings of previous studies may be attributed to the differences in the definition of hyperglycemia, reflecting the challenges in determining an optimal blood glucose (BG) level for LT [18,19]. The optimal BG level was determined according to the most recent and professional guidelines on glycemic control in the present study [20,21,22]. We aimed to determine whether these universally accepted perioperative glucose targets are also applicable in liver transplantation.

In addition to determining the optimal BG level, identifying the optimal timing for glycemic control during LT is also crucial. The BG levels peak during the neohepatic phase and begin to decline 3 h after reperfusion [18,23]. This increase in the BG level may be attributed to the influx of glucose from the grafted liver and peripheral insulin resistance. The BG level gradually decreases after successful LT [24]. Thus, maintaining the BG level within the optimal range, especially during the neohepatic phase, may result in better post-transplantation outcomes.

This study aimed to determine whether controlling the BG levels within the optimal range during the neohepatic phase reduces the incidence of AKI. Furthermore, the severity of AKI, and progression to chronic kidney disease (CKD) were also investigated.

## 2. Materials and Methods

### 2.1. Study Design and Participation

**Figure 1:**
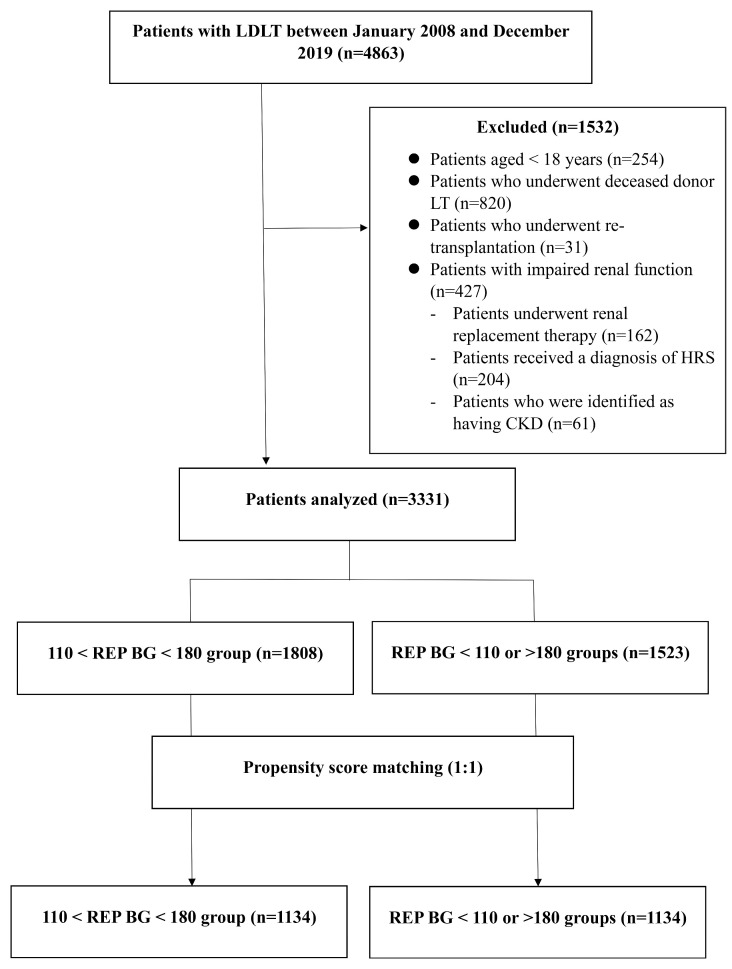
Study flowchart.

The medical records of 4863 consecutive patients scheduled to undergo living donor liver transplantation (LDLT) between January 2008 and December 2019 (Biomedical Research Program of our institution) were retrospectively analyzed. The research protocol was registered at ClinicalTrials.gov and approved by the institutional review board of Asan Medical Center (2020-0675). Because of the retrospective nature of this study, the requirement for written informed consent was waived. The work has been reported in line with the strengthening the reporting of cohort, cross-sectional, and case–control studies in surgery (STROCSS) criteria [25]. The application of the following criteria resulted in the exclusion of 1532 patients: age < 18 years; patients who underwent deceased donor LT; patients who underwent re-transplantation; and impaired renal function, such as CKD or HRS. Thus, 3331 patients were included in the final analysis (Figure 1).

The BG levels during the perioperative phase have conventionally been maintained via inpatient glycemic management owing to the lack of evidence-based guidelines. Recently, the majority of professional guidelines recommend maintaining BG levels below 180 mg/dL [20,21]. Previous studies have set the optimal perioperative target range as 110–180 mg/dL to reduce the incidence of adverse outcomes and prevent hypoglycemia [22]. Therefore, the optimal BG level after reperfusion during LT was set as 110–180 mg/dL in the present study. The patients were divided into two groups based on the BG level during the neohepatic phase of liver transplantation: the optimal BG level after reperfusion (REP BG) group comprising patients with BG levels > 110 mg/dL but <180 mg/dL after reperfusion, and the non-optimal REP BG group comprising patients with BG < 110 mg/dL and >180 mg/dL. BG levels were closely monitored at 30 min to 1 h intervals throughout the intra-operative period to ensure tight glycemic control.

### 2.2. Clinical Data Collection

Baseline demographic data, laboratory results, and postoperative outcomes were extracted from the electronic medical record system of Asan Medical Center. Demographic variables included age, sex, weight, height, body mass index (BMI), and the American Society of Anesthesiologists (ASA) physical status classification.

Pre-operative laboratory data included serum albumin, hemoglobin (Hb), creatinine, and C-reactive protein (CRP). Hypoalbuminemia was defined as serum albumin < 3.5 g/dL. Anemia was defined as Hb < 12 g/dL in females and Hb < 13 g/dL in males.

Additional clinical information included the type of surgery, anesthesia method, and operation time. Surgeries performed within 48 h of hospital admission were classified as early surgery; those performed after 48 h were categorized as late surgery.

### 2.3. Anesthetic Techniques and Surgical Procedures

Standard monitoring, as recommended by the American Society of Anesthesiologists, was commenced before the induction of anesthesia. Anesthetic management and patient care for LT were performed in accordance with the established institutional protocol of our institution, as described previously [26]. Anesthesia was induced via the administration of propofol, fentanyl, midazolam, and rocuronium. Anesthesia was maintained via the administration of inhalation agents and the continuous infusion of fentanyl and rocuronium. Radial and femoral artery cannulation was performed intra-operatively for continuous arterial monitoring and the central venous catheter was inserted into the internal jugular vein for the infusion of fluids. A Swan–Ganz catheter was inserted via a 9Fr introducer sheath to monitor the pulmonary arterial pressure and cardiac output. The native liver of the recipient was dissected to expose the inferior vena cava after separating the portal vein, hepatic artery, and bile duct. After harvesting the native liver, the graft was transplanted into the recipient. The inferior vena cava was clamped partially without veno-venous bypass. An end-to-end anastomosis between the right portal vein of the donor and the portal vein of the recipient was performed after completing hepatic vein anastomosis. Graft reperfusion was performed after completing portal vein anastomosis. Patients with postreperfusion syndrome received norepinephrine (10 mcg) or epinephrine (10 mcg) based on the severity. Continuous infusion of norepinephrine was commenced to achieve a target mean arterial pressure of >70% of the baseline. Sequential anastomosis of the hepatic artery and bile duct was performed after graft reperfusion. The recipients were transferred to the intensive care unit postoperatively without extubation. The detailed surgical procedures for LDLT have been described in previous studies [27].

### 2.4. Immunosuppression Protocol

Immunosuppression protocols used for LT recipients at our institution consisted of interlukin-2 receptor inhibitor on days 0 and 4; an intra-operative bolus of corticosteroid (5–10 mg/kg) according to recipient and graft condition; intravenous or oral calcineurin inhibitor recycling beginning on day 1; and adjunctive mycophenolate mofetil for patient acute cellular rejection, as previously described [28]. Corticosteroids were administered in the form of intravenous methylprednisolone after reperfusion during surgery and rapidly tapered within the first 3 months.

The target 12 h trough concentration of tacrolimus was around 10–15 ng/mL for the first month, 8–10 ng/mL within the first year, 5–8 ng/mL at 2–3 years, 5 ng/mL at 4–5 years, 3–5 ng/mL at 6–10 years, and 2–3 ng/mL after 10 years. When MMF was used for CNI sparing, the target tacrolimus concentration was reduced to half or less. The detailed target trough levels of tacrolimus with and without MMF relative to the post-transplantation period have been summarized previously [29].

### 2.5. Data Collection and Assessment

The electronic medical records of the patients were retrospectively reviewed to extract data regarding the baseline characteristics, laboratory findings, intra-operative variables, and postoperative outcomes. Baseline characteristics included severity of liver disease calculated using the model for end-stage liver disease (MELD)/Na score and Child–Turcotte–Pugh (CTP) score; comorbidities; original disease, such as hepatitis B and C virus, alcoholism, and combined hepatocellular carcinoma; and the graft/recipient weight ratio (GRWR). Pre-operative laboratory data included the hematocrit; platelet count; prothrombin time (PT); and serum creatinine (sCr), albumin, sodium, total bilirubin, aspartate aminotransferase (AST), and alanine aminotransferase (ALT) levels. Baseline sCr levels were collected within 1 week before surgery. In addition, donor information, including age, sex, and pre-operative FC, was also collected. Intra-operative variables included the duration of surgery, volume and type of fluids (crystalloid and colloid), blood products transfused, urine output, total ischemic time, the incidence of post-reperfusion syndrome, and the use of vasopressors (norepinephrine). Massive transfusion was defined as the administration of ≥10 units of packed red blood cells (PRBCs) within 24 h or ≥4 units of PRBCs within 1 h [30]. Total ischemic time was defined as the sum of cold and warm ischemic times. Post-reperfusion syndrome was defined as a reduction of >30% in the systemic mean blood pressure from the baseline for at least 1 min within the initial 5 min of liver reperfusion [31].

Intra-operative measurement of the BG levels was performed by the anesthesiology team during the following phase of LT: pre-anhepatic, anhepatic, and 1 h after graft reperfusion. This 1 h post-reperfusion measurement was performed uniformly in all patients according to institutional protocol. The intra-operative BG level was maintained at <180 mg/dL using the insulin infusion protocol of Portland [32]. The same immunosuppression protocol, which involved the administration of tacrolimus and mycophenolate mofetil as the primary immunosuppressive agents, was used in all cases after LDLT [28]. In addition, BG levels were monitored at 30 min to 1 h intervals during surgery to ensure consistent glycemic control.

After LDLT, all patients underwent scheduled outpatient visits every 3 months during the first year, with laboratory tests including renal function assessment at each visit. Patients were followed until death or their last outpatient visit with available laboratory data. The follow-up duration was defined as the interval from the date of LDLT to the last available renal function assessment within one year after transplantation.

### 2.6. Primary and Secondary Outcomes

The primary outcome was the incidence of AKI in the two groups as determined by the change in sCr according to the Kidney Disease: Improving Global Outcomes (KDIGO) definition (increase in sCr levels of ≥26.5 mmol/L within 48 h or ≥1.5 times the baseline value within 7 postoperative days) before and after matching. Stage 1 AKI was defined as an increase in sCr level of ≥26.5 mmol/L (0.3 mg/dL) or an increase in sCr to 1.5 times the baseline value. Stage 2 AKI was defined as an sCr level of 2.0–2.9 times the baseline value. Stage 3 AKI was defined as an sCr level of ≥3.0 times the baseline value, an increase of ≥353.6 mmol/L (4.0 mg/dL) in the sCr level, or the initiation of renal replacement therapy.

The secondary outcomes included the incidence of severe AKI and CKD. Severe AKI was defined as KIDIGO stage 2 or 3 [33]. The patients were followed for up to 1 year to determine whether AKI progressed to CKD. The incidence of CKD within 1 year post-LT was defined as renal function (assessed by calculating estimated serum glomerular filtration using the Chronic Kidney Disease Epidemiology Collaboration formula (CKD-EPI) of <60 mL/min/1.73 m^2^) of <60 mL/min/1.73 m^2^ for ≥3 months, irrespective of the cause [34]. Data regarding the outcomes, including the incidence of AKI and CKD, were collected from the medical or insurance records of the patients.

### 2.7. Statistical Analysis

All statistical analyses were performed using R statistical software, version 3.6.3 (R Foundation for Statistical Computing, Vienna, Austria). Continuous variables are presented as means ± standard deviations or medians (interquartile ranges), as appropriate. The age; BMI; pre-operative lab data; MELD/Na score; CTP score; amounts of administered crystalloid, colloid, and RBCs administered; urine output; total ischemic time; and GRWR of the patients were compared using Student’s *t*-test. Categorical variables are presented as frequencies and percentages and were analyzed using the chi-squared test or Fisher’s exact test. Multiple logistic regression analysis was performed to identify the factors associated with the incidence of AKI. All variables with a *p*-value of <0.1 in the univariate analysis were included in the multivariate analysis. A *p*-value of <0.05 was considered statistically significant. Weighted logistic regression analyses were employed to evaluate the adjusted odds ratios (ORs) of BG levels after reperfusion to determine their effect on the outcome variables. Multiple logistic regression analysis was performed using the following variables to determine the PS: age, sex, BMI, MELD/Na scores, CTP score, comorbidities, original disease, GRWR, operation time, total ischemic time, donor age, donor sex, pre-operative fatty change, and massive transfusion (Table 1). The patients were matched at a 1:1 ratio using greedy matching algorithms after determining the PSs. The Hosmer–Lemeshow test and C-statistic were used to evaluate model calibration (chi-square statistic = 13.913; df = 8, *p* = 0.084) and model discrimination (0.720), respectively. The absolute standardized mean difference (SMD) was calculated to determine the balance after matching. Categorical and continuous variables were compared using the McNemar test and the paired *t*-test or Wilcoxon signed-rank test, respectively, as appropriate.

## 3. Results

A total of 3331 patients were reviewed and divided into two groups based on the BG levels after reperfusion: the optimal REP BG (*n* = 1808) and the non-optimal REP BG (*n* = 1523) groups (Figure 1).

The final analysis included 1134 patients in each group after PS matching. Table 1 presents the demographic, pre-operative, and intra-operative characteristics of the patients according to the BG level after reperfusion. No statistically significant differences were observed between the two groups after PS matching in terms of any of the parameters outlined in Table 1.

**Figure 2:**
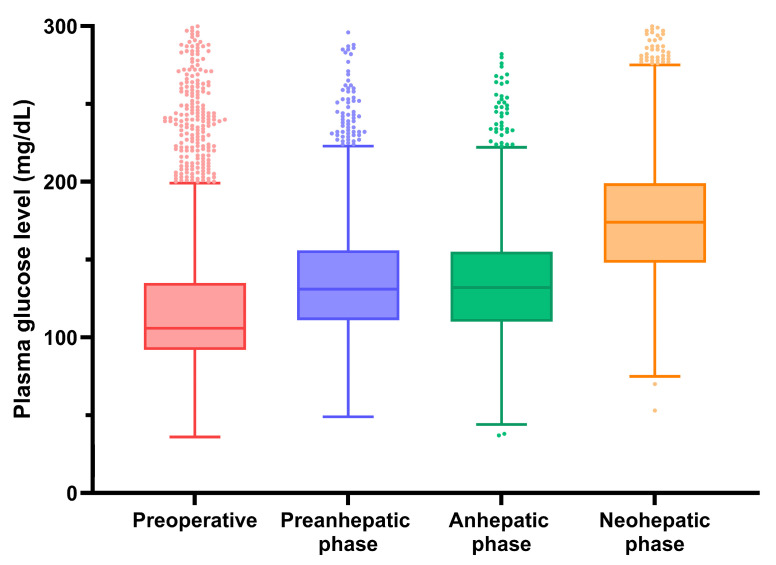
Plasma glucose levels during the perioperative period of LDLT. The figure illustrates the temporal trend of plasma glucose levels measured pre-operatively and at intra-operative phases, including pre-anhepatic, anhepatic, and neohepatic phases.

Figure 2 illustrates the plasma glucose levels measured pre-operatively and intra-operatively.

Multivariable logistic regression analysis showed that the non-optimal REP BG group had a significantly higher risk of AKI compared to the optimal group (OR, 1.25; 95% CI, 1.07–1.47; *p* = 0.007; Table 2).

The CTP scores (OR, 1.14; 95% CI, 1.09–1.19; *p* < 0.001), HCV infection (OR, 1.59; 95% CI, 1.15–2.23; *p* = 0.006), duration of anesthesia (OR, 1.17; 95% CI, 1.13–1.22; *p* < 0.001), and mean tacrolimus trough level (OR, 1.10; 95% CI, 1.07–1.14; *p* < 0.001) were associated with the incidence of AKI. In contrast, GRWR (OR, 0.51; 95% CI, 0.37–0.71; *p* < 0.001) and use of vasopressor (OR, 0.74; 95% CI, 0.62–0.88; *p* < 0.001) were inversely associated with the incidence of AKI.

Table 3 presents the associations between the BG level after reperfusion and postoperative outcomes. Among the 3331 patients who underwent LT, AKI, severe AKI, and progression to CKD were observed in 2223 (66.7%), 994 (29.8%), and 1585 (48.5%) patients, respectively (Table 3). Of those who developed CKD, 46 patients (2.9%) required dialysis within one year, representing 1.4% of the entire cohort. A detailed subgroup analysis of the interaction between optimal REP BG and clinical characteristics on the risk of AKI and CKD is provided in Supplementary Table S1.

**Figure 3:**
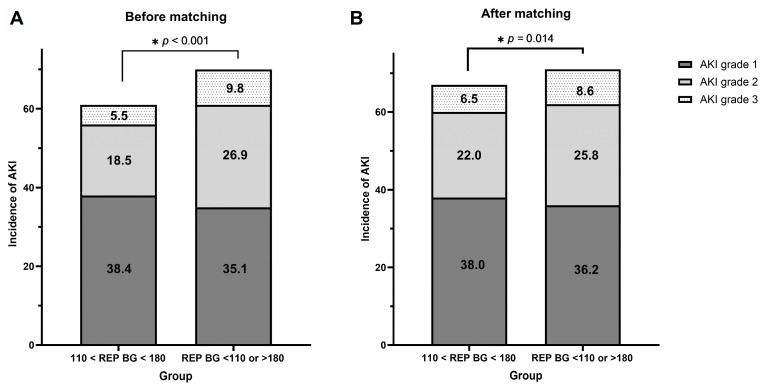
Proportion of patients with acute kidney injury graded according to the Kidney Disease: Improving Global Outcomes (KDIGO) criteria between optimal REP BG (110 mg/dL < REP BG < 180 mg/dL) and non-optimal REP BG (REP BG < 110 mg/dL or >180 mg/dL) groups before (**A**) and after (**B**) propensity score-matching analysis. *: indicating statistical significance.

The prevalence of AKI in the optimal REP BG group was lower than that in the non-optimal REP BG group, and significant differences were observed between the two groups before and after PS matching (62.4% vs. 71.8%, *p* < 0.001, and 66.5% vs. 70.6%, *p* = 0.032, respectively). The severity of AKI according to the KDIGO definition was compared between the two groups. The severity of AKI in the non-optimal REP BG group was significantly higher than that in the optimal REP BG group in the crude and post-PS matching cohorts (*p* < 0.001 and *p* = 0.014, respectively). Figure 3 illustrates the prevalence of AKI severity in the two groups before (Figure 3A) and after PS matching (Figure 3B).

The incidence of AKI was independently associated with blood glucose levels after reperfusion before and after propensity score-matching (OR, 1.53; 95% CI, 1.32–1.78; *p* < 0.001 and OR, 1.21; 95% CI, 1.02–1.45; *p* = 0.037, respectively; Table 3). Similarly, the incidence of severe AKI and progression to CKD was significantly higher in the non-optimal REP BG group than in the optimal REP BG group before and after propensity score-matching (severe AKI: OR, 1.83; 95% CI, 1.58–2.13; *p* < 0.001 and OR, 1.32; 95% CI, 1.11–1.58; *p* = 0.002; CKD: OR, 1.28; 95% CI, 1.11–1.46; *p* = 0.001 and OR, 1.19; 95% CI, 1.01–1.41; *p* = 0.039, respectively).

## 4. Discussion

A retrospective analysis of 3331 patients who underwent LDLT was performed to determine the impact of optimal glycemic control after reperfusion on the incidence of AKI post-LT. Maintenance of optimal BG levels after reperfusion, defined as 110 mg/dL < REP BG < 180 mg/dL, was independently associated with a lower incidence of AKI post-LT. This finding was validated via PS matching and multivariable regression analysis. In addition, maintenance of optimal BG levels after reperfusion showed significant associations with reduced severity of postoperative AKI, and progression to CKD.

Importantly, patients whose BG levels were tightly maintained within this target range during the neohepatic phase exhibited not only a lower incidence but also a reduced severity of AKI, along with a decreased risk of progression to CKD. These findings underscore the potential clinical benefit of strict glycemic control during the immediate post-reperfusion period in LDLT recipients.

Glucose instability, particularly hyperglycemia, is associated with the incidence of adverse postoperative outcomes [35]. However, the association of the perioperative glucose levels with the incidence of AKI has been investigated in relatively small cohorts of patients [17]. No large-scale studies of perioperative glucose levels, especially the BG levels after reperfusion, and the incidence of AKI following LDLT have been conducted. To the best of our knowledge, the present study is the first large retrospective analysis to investigate the association between the BG levels after reperfusion and the incidence of AKI following LDLT.

Optimal glycemic control after reperfusion plays an important role in reducing the incidence of AKI post-LT; however, the exact mechanism remains unclear. Hyperglycemia may induce kidney damage by producing inflammatory cytokines and reactive oxygen species [36,37]. In addition, hypoglycemia may also cause kidney injury by elevating the C-reactive protein and pro-inflammatory cytokine levels [38]. Thus, maintaining optimal glycemic levels after reperfusion, rather than merely preventing hyperglycemia, may mitigate renal damage and reduce the incidence of AKI.

Yoo et al. report that the increased glucose variability, rather than hyperglycemia (BG of >200 mg/dL), in the intra-operative and early postoperative glucose levels post-LT is associated with an increased incidence of AKI [17]. This disparity may be attributed to the variations in the glucose target level. Yoo et al. [17] defined normoglycemia as BG levels of 80–200 mg/dL, a broader spectrum compared with that used in the present study. Consequently, patients with more adverse outcomes were included in the normoglycemia group. Another contributing factor may be the timing of BG level measurement. Unlike previous studies that examined BG levels during the perioperative and early postoperative phases [17], the present study focused solely on the BG levels after reperfusion, a period during which BG level variability and the risk of hyperglycemia are most prominent [18]. Considering other studies that suggest peak glucose levels influence the development of AKI [39,40], hyperglycemia during the neohepatic phase may have the most detrimental effect in LT. Additionally, a previous study has demonstrated that post-reperfusion syndrome during LT may contribute to the development of intra-operative stress hyperglycemia [41], eventually leading to adverse clinical outcomes.

In addition to the difference in the incidence of AKI, a significant difference was observed in the severity of AKI between the two groups before and after matching. Logistic regression analysis revealed that the BG levels after reperfusion showed a significant association with the severity of AKI. Severe AKI is associated with an increased likelihood of progression to CKD [42]. The present study also revealed that maintenance of an optimal BG level after reperfusion showed a significant association with the decreased incidence of CKD within one-year post-LT.

Multivariate logistic regression analysis revealed that a high CTP score, HCV infection, a prolonged duration of surgery, elevated mean of tacrolimus trough level, and low GRWR showed significant associations with a higher incidence of AKI. In contrast, our study revealed that the intra-operative use of vasopressor was associated with a reduced incidence of AKI. Consistent with the findings of numerous previous studies, the incidence of AKI was associated with the severity of the underlying liver disease, as assessed by a higher CTP score, in the present study [14,15]. Viral hepatitis induces glomerular changes accompanied by hemodynamic instability, which may play a crucial role in the incidence of AKI post-LT. The present study suggested that HCV infection may be a risk factor for the development of AKI. Prolonged duration of surgery may be a risk factor for the incidence of AKI owing to the reduction in renal perfusion resulting from sympathetic renal artery contraction, potentially leading to kidney damage [43]. A previous study reported a positive correlation between the mean tacrolimus trough levels and renal impairment following liver transplantation, attributed to its known renal toxicity [44]. A low GRWR predisposes patients to persistent portal hypertension and a hyperdynamic state, potentially disrupting the balance between the vasodilator and vasoconstrictor factors, thereby contributing to the incidence of AKI post-LT [13]. Lastly, norepinephrine was continuously infused to maintain a target mean arterial pressure of at least 70% of baseline. However, given the common occurrence of reduced systemic vascular resistance (SVR) in LT recipients [45], early application of norepinephrine was employed to maintain adequate perfusion in our institution. In our opinion, this strategy may have improved renal perfusion and subsequently reduced the incidence of AKI. While glycemic control remains a priority, our results indicate that operative time and graft-related factors, such as GRWR, are also important modifiable contributors to AKI risk. Thus, a comprehensive perioperative approach addressing both metabolic and surgical factors may help minimize postoperative kidney injury.

The present study has some limitations. First, undocumented factors and potential bias may have influenced the results of the present study owing to its retrospective nature. Multivariable regression analysis and propensity score matching was performed to mitigate this bias. Second, as this study was conducted at a facility with a high volume of surgeries (300–400 LDLT performed since 2010), these findings may not apply to smaller institutions, as the clinical course of LT may be influenced by the volume of cases [46]. Lastly, a single measurement of BG levels after reperfusion may have potentially underestimated the incidence of glucose instability. Continuous monitoring of BG levels throughout the post-reperfusion period would facilitate a more comprehensive understanding of glucose dynamics and potential instability events; however, a single measurement was sufficient to determine its association with the postoperative incidence of AKI.

## 5. Conclusions

In conclusion, maintaining optimal BG levels after reperfusion resulted in a significantly lower incidence of AKI and a reduced risk of progression to CKD within 1 year post-LDLT. Thus, maintaining the BG levels after reperfusion may aid in mitigating the incidence of AKI following LDLT.

## Figures and Tables

**Table 1:** Patient characteristics and perioperative variables before and after propensity score matching.

	Before PSM				After PSM			
	Optimal REP BG (*n* = 1808)	Non-Optimal REP BG (*n* = 1523)	*p*	SMD	Optimal REP BG (*n*= 1134)	Non-Optimal REP BG (*n* = 1134)	*p*	SMD
Demographic characteristics								
Age, years	53.7 ± 8.2	52.9 ± 8.8	0.007	0.094	53.4 ± 8.2	53.3 ± 8.7	0.754	−0.013
Sex, male	1095 (71.9%)	1369 (75.7%)	0.012	0.087	820 (72.3%)	821 (72.4%)	0.963	−0.002
BMI, kg/m^2^	24.6 ± 3.6	24.2 ± 3.3	0.007	0.094	24.4 ± 3.3	24.5 ± 3.5	0.603	0.022
Pre-operative variables								
DM	463 (30.4%)	322 (17.8%)	<0.001	0.298	266 (23.5%)	298 (26.3%)	0.120	−0.065
HTN	238 (15.6%)	312 (17.3%)	0.207	0.044	179 (15.8%)	181 (16.0%)	0.909	−0.005
MELD_Na score	16.6 ± 7.8	13.2 ± 7.2	<0.001	0.457	15.2 ± 7.2	15.0 ± 7.8	0.468	−0.031
CTP	8.5 ± 2.2	7.4 ± 2.2	<0.001	0.493	8.1 ± 2.1	8.0 ± 2.3	0.247	−0.005
HCC	744 (48.9%)	1044 (57.7%)	<0.001	0.179	592 (52.2%)	579 (51.1%)	0.585	0.023
Original disease								
HBV	917 (60.2%)	1188 (65.7%)	0.001	0.144	705 (62.2%)	695 (61.3%)	0.666	0.018
HCV	122 (8.0%)	99 (5.5%)	0.003	0.101	76 (6.7%)	78 (6.9%)	0.867	−0.007
Alcoholic	320 (21.0%)	291 (16.1%)	<0.001	0.127	225 (19.8%)	231 (20.4%)	0.753	−0.013
GRWR, %	1.1 ± 0.3	1.1 ± 0.2	<0.001	0.248	1.1 ± 0.2	1.1 ± 0.3	0.493	−0.029
Pre-operative lab data								
Hematocrit, %	31.4 ± 5.9	33.5 ± 6.4	<0.001	0.335	32.0 ± 6.0	32.1 ± 6.3	0.930	0.004
Platelets, ×10^3^/µL	67.6 ± 45.2	82.4 ± 55.3	<0.001	0.294	78.3 ± 71.4	77.0 ± 53.7	0.628	0.020
Prothrombin time, INR	1.6 ± 0.6	1.4 ± 0.6	<0.001	0.261	1.5 ± 0.5	1.5 ± 0.7	0.264	0.047
Creatinine, mg/dL	0.8 ± 0.2	0.8 ± 0.2	0.153	0.050	0.8 ± 0.2	0.8 ± 0.2	0.091	0.071
Albumin, g/dL	3.0 ± 0.6	3.2 ± 0.6	<0.001	0.389	3.1 ± 0.5	3.1 ± 0.6	0.276	0.046
Sodium, mmol/L	137.2 ± 5.4	139.0 ± 4.3	<0.001	0.360	137.9 ± 4.9	138.3 ± 4.7	0.069	0.076
Total bilirubin, mg/dL	5.8 ± 8.8	3.8 ± 6.9	<0.001	0.261	5.0 ± 8.0	4.9 ± 8.1	0.834	0.006
AST, IU/L	69.3 ± 175.7	57.6 ± 114.4	0.025	0.079	65.9 ± 132.9	65.4 ± 137.4	0.926	0.004
ALT, IU/L	53.2 ± 215.7	50.5 ± 171.1	0.699	0.014	50.7 ± 175.9	57.4 ± 201.5	0.402	0.035
HbA1c, %	5.6 ± 1.2	5.8 ± 1.4	<0.001	0.143	5.7 ± 1.3	5.8 ± 1.4	0.429	0.037
Intra-operative variables								
Duration of anesthesia, min	866.7 ± 150.2	830.8 ± 142.7	<0.001	0.245	851.2 ± 145.5	850.0 ± 149.3	0.847	−0.008
Crystalloid infusion, mL	7902.1 ± 4015.8	7343.4 ± 4126.3	<0.001	0.137	7515.8 ± 3545.8	7636.8 ± 4762.4	0.493	0.029
Colloid infusion, mL	4285.3 ± 3083.0	3541.2 ± 3303.5	<0.001	0.233	4036.4 ± 3988.3	4001.5 ± 3921.1	0.812	0.010
Massive transfusion	734 (48.2%)	437 (24.2%)	<0.001	0.516	432 (38.1%)	419 (37.0%)	0.573	−0.024
Urine output, mL	1892.4 ± 984.2	2065.1 ± 1134.9	<0.001	0.163	2006.8 ± 1316.5	2014.5 ± 1089.0	0.879	0.006
Total ischemic time, min	127.4 ± 32.0	127.0 ± 37.8	0.767	0.010	126.2 ± 31.0	127.5 ± 34.0	0.336	0.040
Post-reperfusion syndrome	934 (61.3%)	986 (54.5%)	<0.001	0.138	676 (59.6%)	680 (60.0%)	0.864	0.007
Use of vasopressor, %	992 (65.1)	1209 (66.9)	0.299	0.037	743 (65.5)	770 (67.9)	0.247	0.051
Postoperative variable								
Mean cTAC, ng/mL	9.9 ± 2.7	9.3 ± 2.8	<0.001	0.212	6.5 ± 3.2	6.7 ± 3.3	0.159	0.069
Donor characteristics								
Age, years	28.7 ± 8.4	28.3 ± 8.3	0.170	0.048	28.5 ± 8.6	28.5 ± 8.6	0.660	−0.019
Sex, male	1043 (68.5%)	1238 (68.5%)	0.995	<0.001	773 (68.2%)	773 (68.2%)	0.964	−0.002
Pre-operative FC	6.6 ± 8.3	6.8 ± 8.2	0.515	0.023	6.6 ± 8.2	6.8 ± 823	0.559	0.025

Results are presented as mean ± SD, *n* (%). PSM, propensity score matching; REP BG; reperfusion blood glucose; SMD, standardized mean difference; BMI, body mass index; DM, diabetes mellitus; HTN, hypertension; MELD, model for end-stage liver disease; CTP, Child–Pugh score; HBV, hepatitis B virus; HCV, hepatitis C virus; HCC, hepatocellular carcinoma; GRWR, graft/recipient weight ratio; cTAC, mean tacrolimus trough level within the first 7 postoperative days; FC, fatty liver change.

**Table 2:** Logistic regression analyses of the risk factors associated with AKI.

	Univariate			Multivariate		
	OR	95% CI	*p*	OR	95% CI	*p*
Pre-operative variables						
Age, years	1.00	0.99–1.01	0.744			
Sex, male	0.91	0.77–1.07	0.262			
DM	1.18	1.00–1.41	0.055			
HTN	1.06	0.87–1.29	0.556			
MELD_Na score	1.04	1.03–1.05	<0.001			
CTP	1.18	1.14–1.22	<0.001	1.14	1.09–1.19	<0.001
HBV	0.91	0.78–1.05	0.200			
HCV	1.59	1.16–2.19	0.004	1.59	1.15–2.23	0.006
Alcoholic	1.06	0.88–1.28	0.553			
HCC	0.72	0.62–0.83	<0.001			
GRWR (%)	0.50	0.37–0.67	<0.001	0.51	0.37–0.71	<0.001
Intra-operative variables						
Non-optimal REP BG *	1.54	1.33–1.79	<0.001	1.25	1.06–1.47	0.007
Duration of anesthesia, hours	1.24	1.20–1.29	<0.001	1.17	1.13–1.22	<0.001
Cold ischemic time, hours	1.00	1.00–1.00	0.657			
Warm ischemic time, hours	1.01	1.01–1.02	<0.001			
Massive transfusion	2.03	1.73–2.39	<0.001			
Post-reperfusion syndrome	0.87	0.75–1.01	0.059			
Use of vasopressor	0.62	0.53–0.73	<0.001	0.74	0.62–0.88	<0.001
Donor-related variables						
Donor age, years	1.01	1.00–1.01	0.191			
Donor sex, male	1.04	0.89–1.21	0.650			
Pre-operative FC	0.99	0.99–1.00	0.227			
Postoperative variables						
Mean cTAC	1.10	1.07–1.13	<0.001	1.10	1.07–1.14	<0.001

* Reference: optimal REP BG (110 mg/dL < BG < 180 mg/dL). AKI, acute kidney injury; OR, odds ratio; CI, confidence interval; REP BG; reperfusion blood glucose; DM, diabetes mellitus; HTN, hypertension; MELD, model for end-stage liver disease; CTP, Child–Pugh score; HBV, hepatitis B virus; HCV, hepatitis C virus; HCC, hepatocellular carcinoma; GRWR, graft/recipient weight ratio; cTAC, mean tacrolimus trough level within the first 7 postoperative days.

**Table 3:** Clinical outcomes before and after propensity score-matching.

Crude					Propensity Score-Matching			
	Event/N (%)	OR	95% CI	*p*	Event/N (%)	OR	95% CI	*p*
AKI								
Optimal REP BG	1129/1808 (62.4)	1 (reference)			754/1134 (66.5)	1 (reference)		
Non-optimal REP BG	1094/1523 (71.8)	1.53	1.32–1.78	<0.001	801/1134 (70.6)	1.21	1.02–1.45	0.037
Severe AKI								
Optimal REP BG	435/1808 (24.1)	1 (reference)			323/1134 (28.5)	1 (reference)		
Non-optimal REP BG	559/1523 (36.7)	1.83	1.58–2.13	< 0.001	391/1134 (34.5)	1.32	1.11–1.58	0.002
CKD								
Optimal REP BG	814/1780 (45.7)	1 (reference)			512/1132 (45.2)	1 (reference)		
Non-optimal REP BG	772/1490 (51.8)	1.28	1.11–1.46	0.001	561/1131 (49.6)	1.19	1.01–1.41	0.039

REP BG, reperfusion blood glucose; PSM, propensity score-matching; OR, odds ratio; CI, confidence interval; AKI, acute kidney injury, REP BG, blood glucose after reperfusion.

## Data Availability

The datasets used and/or analyzed during the current study are available from the corresponding author on reasonable request.

## Supplementary Materials

The following supporting information can be downloaded at: https://www.mdpi.com/article/10.3390/medicina61081325/s1, Table S1: Subgroup analysis of interaction between optimal REP BG and clinical characteristics on the risk of AKI and CKD.

## Abbreviations

The following abbreviations are used in this manuscript:

AKI	Acute kidney injury
ALT	Alanine aminotransferase
AST	Aspartate aminotransferase
BG	Blood glucose
BMI	Body mass index
CI	Confidence interval
CKD	Chronic kidney disease
CKD-EPI	Chronic Kidney Disease Epidemiology Collaboration
CNI	Calcineurin inhibitor
CTP	Child–Turcotte–Pugh score
DM	Diabetes mellitus
FC	Fatty change
GRWR	Graft-to-recipient weight ratio
HBV	Hepatitis B virus
HCC	Hepatocellular carcinoma
HCV	Hepatitis C virus
HTN	Hypertension
INR	International normalized ratio
KDIGO	Kidney Disease: Improving Global Outcomes
LDLT	Living donor liver transplantation
LT	Liver transplantation
MMF	Mycophenolate mofetil
OR	Odds ratio
*p*	*p*-value
PRBC	Packed red blood cell
PSM	Propensity score matching
PT	Prothrombin time
REP BG	Reperfusion blood glucose
SCr	Serum creatinine
SMD	Standardized mean difference
STROCSS	Strengthening the Reporting of Cohort, Cross-sectional and Case-control Studies in Surgery
SVR	Systemic vascular resistance
cTAC	Mean tacrolimus trough level
