# Supplementary material for: Impact of Glycemic Control After Reperfusion on the Incidence of Acute Kidney Injury Following Living Donor Liver Transplantation: A Propensity Score-Matched Analysis
Source: Medicina (Kaunas). 2025 Jul 23;61(8):1325. doi: 10.3390/medicina61081325 (PMC12387770; doi:10.3390/medicina61081325)
Supplement: Supplementary file 1 [file medicina-61-01325-s001.zip › medicina-3756298-supplementary.pdf]

Supplementary Table 1. Subgroup analysis of interaction between optimal REP BG and clinical characteristics on the risk of AKI and CKD

Subgroup variables	AKI			CKD		
	P for interaction	Odds Ratio	P-value	P for interaction	Odds Ratio	P-value
Age	0.463	1.01	0.99–1.02	0.436	0.99	0.98–1.01
Sex	0.724	0.94	0.67–1.32	0.983	1.00	0.71–1.41
MELD	0.037	1.02	1.00–1.04	0.101	1.01	1.00–1.03
GRWR	0.340	1.35	0.73–2.49	0.718	0.91	0.51–1.63
Massive transfusion	0.160	1.27	0.91–1.77	0.160	0.79	0.58–1.09
Diabetes mellitus	0.152	0.79	0.58–1.08	0.760	1.06	0.75–1.50
Hypertension	0.217	0.79	0.56–1.11	0.556	0.88	0.59–1.31

Odds ratios and P-values reflect the multiplicative interaction terms from multivariable logistic regression models.

AKI, acute kidney injury; CKD, chronic kidney disease; GRWR, graft-to-recipient weight ratio; MELD, model for end-stage liver disease.
